# Clinical Characteristics, Antimicrobial Resistance, Virulence Genes and Multi-Locus Sequence Typing of Non-Typhoidal *Salmonella* Serovar Typhimurium and Enteritidis Strains Isolated from Patients in Chiang Mai, Thailand

**DOI:** 10.3390/microorganisms11102425

**Published:** 2023-09-28

**Authors:** Songphon Buddhasiri, Chutikarn Sukjoi, Arishabhas Tantibhadrasapa, Panupon Mongkolkarvin, Pattarapon Boonpan, Thanakorn Pattanadecha, Nattamon Onton, Touch Laisiriroengrai, Sunatcha Coratat, Banyong Khantawa, Surapun Tepaamorndech, Kwanjit Duangsonk, Parameth Thiennimitr

**Affiliations:** 1 Department of Veterinary Biosciences and Veterinary Public Health, Faculty of Veterinary Medicine, Chiang Mai University, Chiang Mai 50100, Thailand; songphon.bu@cmu.ac.th; 2 Department of Microbiology, Faculty of Medicine, Chiang Mai University, Chiang Mai 50200, Thailand; 3 Diagnostic Laboratory, Maharaj Nakorn Chiang Mai Hospital, Faculty of Medicine, Chiang Mai University, Chiang Mai 50200, Thailand; 4 Department of Microbiology, Faculty of Medicine, Chulalongkorn University, Bangkok 10330, Thailand; 5 Research Center of Microbial Diversity and Sustainable Utilization, Chiang Mai University, Chiang Mai 50100, Thailand; 6 Center of Multidisciplinary Technology for Advanced Medicine, Faculty of Medicine, Chiang Mai University, Chiang Mai 50200, Thailand

**Keywords:** non-typhoidal *salmonellosis*, acute gastroenteritis, multidrug-resistant *Salmonella*, clinical isolated *Salmonella*

## Abstract

Non-typhoidal *salmonellosis* (NTS) caused by ingesting *Salmonella enterica* contaminated food or drink remains a major bacterial foodborne disease. Clinical outcomes of NTS range from self-limited gastroenteritis to life-threatening invasive NTS (iNTS). In this study, we isolated *Salmonella* spp. from the stool and blood of patients hospitalized at Maharaj Nakorn Chiang Mai Hospital, Chiang Mai, Thailand, between 2016–2021 (a total of 395 cases). Then, serovar Typhimurium and Enteritidis were identified and further characterized by multiplex PCR, and multi-locus sequence typing. Our data show that multidrug resistance (MDR) sequence type 34 (ST34) and ST11 are the predominant sequence types for serovars Typhimurium and Enteritidis, respectively. Most *S. Typhimurium* ST34 lacks *spvB*, and most *S. Enteritidis* ST11 harbor *sseI*, *sodCI*, *rpoS* and *spvB* genes. NTS can be found in a wide range of ages, and anemia could be a significant factor for *S. Typhimurium* infection (86.3%). Both *S. Typhimurium* (6.7%) and *S. Enteritidis* (25.0%) can cause iNTS in immunocompromised patients. *S. Typhimurium* conferred MDR phenotype higher than *S. Enteritidis* with multiple antibiotic resistance indexes of 0.22 and 0.04, respectively. Here, we characterized the important *S. Typhimurium*, *S. Enteritidis*, and human clinical factors of NTS within the region.

## 1. Introduction

One of the most frequent and impactful gastrointestinal infections on both human health and economic loss is acute nontyphoidal salmonellosis (NTS) [1,2]. Over 1.35 million cases per year of NTS have been reported in the United States alone [3]. The cause of NTS is *Salmonella enterica* which comprises more than 2500 serovars [4,5]. Among all serovars, two important serovars belonging to *S. enterica* subspecies I that are implicated in the epidemic of NTS worldwide are Typhimurium (*S. Typhimurium*) and Enteritidis (*S. Enteritidis*) [6]. Various clinical manifestations of NTS infections range from self-limited diarrhea to life-threatening invasive bacteremia [7]. Children and individuals with human immunodeficiency virus (HIV) infection are at a high risk of developing invasive NTS (iNTS) [8,9,10]. Throughout the last few decades, the rate of antibiotic resistance and the number of newly discovered resistance determinants in Salmonella have significantly increased [11,12,13]. Multidrug-resistant *Salmonella* strains have become a significant problem in human and animal healthcare systems, and also in the food industry [14].

Multi-locus sequence typing (MLST) has been commonly used to determine Salmonella sequence type (ST) for epidemiological studies [5]. Over time, several ST of *S. Typhimurium* became predominant in human and animal samples, particularly ST34 and ST19, which are the most common STs in Asia [15,16]. Over the past two decades, ST34 emergence has increased significantly as supporting data showed its spread through Europe, North America, Asia, and Australia [17,18,19]. Importantly, ST34 is often associated with multidrug resistance (MDR) phenotype to several antibacterial agents such as ampicillin, chloramphenicol, streptomycin, sulbactam, and tetracycline and extends to other antibacterial agents [20,21]. In contrast, ST19 isolates resisted fewer antibacterial classes [22]. The continuous rise in MDR *S. Typhimurium* has significantly challenged the current therapeutic methods to control or prevent this important foodborne illness. 

*S. Enteritidis* typically causes acute gastrointestinal symptoms (nausea, vomiting, fever with diarrhea), like other non-typhoidal *Salmonella.* However, invasive strains of *S. Enteritidis* have emerged as a leading cause of bloodstream and other invasive infections globally [23,24]. *S. Enteritidis* ST11 is one of the most common ST among *S. Enteritidis* isolates in food, animals, and humans in many countries. Previous studies identified that ST11 is associated with iNTS cases reported in Asia and Africa [25,26]. Besides causing serious infections like septicemia, MDR *S. Enteritidis* is also resistant to commonly prescribed antibacterial drugs [27]. Collectively, these data indicate an urgent need to find alternative ways of treating MDR *S. Enteritidis*.

Host factors such as immune status, gut microbiome, and bacterial virulence factors play a significant role in acute NTS [16,28]. Several virulence factors are crucial for *Salmonella* as they enable it to replicate and proliferate inside the host. Major virulence factors that play a critical role in clinical outcomes of *S. Typhimurium* and *S. Enteritidis* infections including *spvB*, *sseI*, *sodCI*, and *rpoS* have been previously reported [29]. The plasmid-derived *spvB* is essential for encoding *Salmonella* cytotoxin that can rearrange the host cell cytoskeleton for increasing *Salmonella* invasion [30]. The Salmonella *sseI* gene encodes effector protein SseI that allows *Salmonella* to live inside the host cells and is essential for the systemic phase of *Salmonella* infection [31]. The *sseI* gene is associated with bacterial systemic spreading and chronic infection in mice by inhibiting the migration of macrophages and dendritic cells [32]. The *sodCI* gene encodes a phagocytic superoxide dismutase to decrease exogenous oxidative damage from the host cells [33]. The *rpoS* gene has a regulatory function important for Salmonella stress response that increases survival of bacteria inside the host cell [34]. The importance of analyzing the microbiological factors of NTS and clinical features of the patients from whom they were isolated has been demonstrated elsewhere [29]. However, the study of Salmonella virulence genes, serovar, sequence type, and clinical outcomes in human NTS in Chiang Mai, Thailand, is still under investigation. 

## 2. Materials and Methods

### 2.1. Study Design and Clinical Data Collection

A retrospective cross-sectional study was conducted at Maharaj Nakorn Chiang Mai Hospital (MNCMH) in Chiang Mai, Thailand, from October 2016 to September 2021. The MNCMH is a part of the Faculty of Medicine, Chiang Mai University, and is a super-tertiary hospital that receives referred cases throughout Chiang Mai and the nearby provinces. Stool and blood cultures positive for *Salmonella* spp. from the Central Diagnostic Laboratory of MNCMH were received on Salmonella Shigella (SS) agar (Oxoid Limited, Hampshire, UK). In total, 395 isolates were collected in this study. Demographic and clinical data of each patient was collected from the computer-based medical record system. Age, gender, presence of sepsis, hematocrit (Hct) level, and white blood cell count (leukocyte, neutrophil, and lymphocyte) of each patient where *Salmonella* spp. was detected and isolated from, upon admission to the hospital, were recorded. Sepsis was defined as having at least two of the following systemic inflammatory response syndrome (SIRS) criteria: heart rate more than 90 beats per minute, respiratory rate more than 20 breaths per minute, body temperature more than 38 °C or less than 36 °C, and total white blood cells count more than 12,000 or less than 4000/mm^3^ or having band neutrophils more than or equal to 10%. 

### 2.2. Salmonella Serotyping by a Multiplex Polymerase Chain Reaction

A total of 395 clinically isolated non-typhoidal *Salmonella* strains were serotyped using a multiplex polymerase chain reaction (PCR) which targeted *Salmonella* spp., *S. Typhimurium*, and *S. Enteritidis* as previously described [29]. The sequences of primer pairs used in this study are presented in Table 1. In brief, the bacterial genomic DNA was extracted using a boiling method. The bacterial suspension was boiled at 100 °C for 5 min and centrifuged at high speed for 1 min to collect the genomic DNA in the supernatant and used as a DNA template [35]. The quality and quantity of the extracted DNA were assessed by the NanoDrop 2000/2000c Spectrophotometers (Thermo Fisher Scientific, Waltham, MA, USA). Then, PCR amplification was performed in a total reaction volume of 25 µL using DreamTaq Green PCR Master Mix (Thermo Fisher scientific, Waltham, MA, USA) with 40 ng of template DNA, and a final concentration of each primer was 0.2 μM. PCRs were performed using a peqSTAR thermal cycler (Peqlab Biotechnologie GmbH, Erlangen, Germany) with the following conditions: an initial denaturation of 95 °C for 5 min; followed by 35 cycles of 95 °C for 30 s, 56 °C for 45 s, and 72 °C for 45 s; and a final extension at 72 °C for 10 min. PCR products were run on a 2.5% agarose gel stained with SYBR Safe DNA Gel Stain (Thermo Fisher Scientific, Waltham, MA, USA), and both positive and negative controls were included in each run. 

### 2.3. Antimicrobial Susceptibility Testing

Antimicrobial susceptibility was tested by the disk diffusion method following M100 and M02-A11 protocol of the Clinical and Laboratory Standards Institute (CLSI) [38,39]. Briefly, a few fresh colonies were resuspended in 3 mL phosphate-buffered saline (PBS) and standardized the turbidity equivalent to a 0.5 McFarland standard. The bacterial suspension was spread over the entire Mueller Hinton agar surface (Oxoid Limited, Hampshire, UK) agar plate. The antimicrobial disks were placed, and plates were then incubated at 37 °C for 16–18 h. The diameter of the zone of inhibition was measured in millimeters using a sliding caliper. The interpretation was based on the CLSI guidelines. The clinical breakpoints were used as interpretive criteria, as suggested by the European Committee on Antimicrobial Susceptibility Testing (EUCAST). Then, a “non-susceptible” to an antimicrobial agent was assigned when it tested resistant, intermediate, or non-susceptible [40]. The following antimicrobials (Oxoid, Basingstoke, UK) and disk potencies (µg) were used: amikacin (30), ampicillin (10), amoxicillin/clavulanic acid (30), azithromycin (15), aztreonam (30), cefepime (30), cefotaxime (30), cefoxitin (30), ceftazidime (30), ceftriaxone (30), cefuroxime (30), cephazolin (30), ciprofloxacin (5), doripenem (10), doxycycline (30), ertapenem (10), imipenem (10), levofloxacin (5), meropenem (10), nalidixic acid (30), piperacillin/tazobactam (110), streptomycin (10), tetracycline (30), and trimethoprim/sulfamethoxazole (25). Reference strains *Escherichia coli* ATCC 25922 and *Pseudomonas aeruginosa* ATCC 27853 were used as quality control. Non-susceptible to any one of cefotaxime, ceftazidime, and aztreonam were screened for extended-spectrum β-lactamase (ESBL) production by a double disc synergy test (DDST) between ceftazidime, cefotaxime and amoxicillin with clavulanic acid. The 13 antimicrobial classes and their agents are demonstrated in Supplementary Table S1. 

### 2.4. Determination of Multiple Antibiotic Resistance (MAR) Index

The multiple antibiotic resistance (MAR) index, the ratio of the number of antibiotics that an isolate was resistant to, and the total number of antibiotics tested for each isolate, (120 for *S. Typhimurium* and 28 for *S. Enteritidis*) was calculated as previously described [41,42]. A MAR index > 0.2 indicates that isolates are from high-risk contaminated sources with frequent use of antibiotics, whereas values ≤ 0.2 indicate that isolates are from sources that have been less exposed to antibiotic use. 

### 2.5. Detection of Salmonella Virulence Genes

The important virulence genes for Salmonella (*spvB*, *sseI*, *sodCI*, and *rpoS*) were detected by PCR with the primer pairs listed in Table 1. In summary, one fresh colony of *S. Typhimurium* or *S. Enteritidis* was picked and resuspended in 50 μL UltraPure DNase/RNase-Free Distilled Water (Thermo Fisher Scientific, Waltham, MA, USA). The bacterial genomic DNA was extracted using the boiling method as mentioned above. PCR was performed in 50 μL of DreamTaq Green PCR Master Mix (Thermo Fisher Scientific, Waltham, MA, USA) following the manufacturer’s instructions and the previous study [29].

### 2.6. Identification of Salmonella Sequence Type by Multi-Locus Sequence Typing (MLST)

The sequence type (ST) of *Salmonella enterica* isolates (n = 30) was identified by a multi-locus sequence typing (MLST) as previously described [43]. In brief, the seven housekeeping genes of *Salmonella,* including *aroC*, *dnaN*, *hemD*, *hisD*, *purE*, *sucA*, and *thrA* were amplified using specific primers listed in Table 1 followed by purification with a GeneJET PCR Purification Kit (Thermo Fisher scientific, Waltham, MA, USA) before being sequenced (1st Base, Axil Scientific, Singapore). Sequences were compared with existing alleles available in the PubMLST *Salmonella* genome database (https://pubmlst.org/salmonella/) (accessed on 2 December 2021) to identify an allelic profile and ST of each isolate. The sequences for Salmonella seven housekeeping genes (*aroC*, *dnaN*, *hemD*, *sucA*, *thrA*, *purE*, and *hisD*) of the isolates in this study were deposited to the GenBank database under the accession numbers OR504765 to OR504974.

### 2.7. Statistical Analysis

Patient, clinical, and laboratory data were recorded in Microsoft Excel 2017 and analyzed with the Statistical Package for Social Sciences, SPSS (version 25, IBM Corp., Somers, NY, USA). Mann-Whitney U test was used for comparison of age. Pearson’s chi-squared and Fisher’s exact test were used to test association. 

## 3. Results

### 3.1. Salmonella Enterica Serovar Typhimurium and Enteritidis Strains Were Identified from Hospitalized Patients with Stool or Blood Cultures Positive for Salmonella

**Figure 1:**
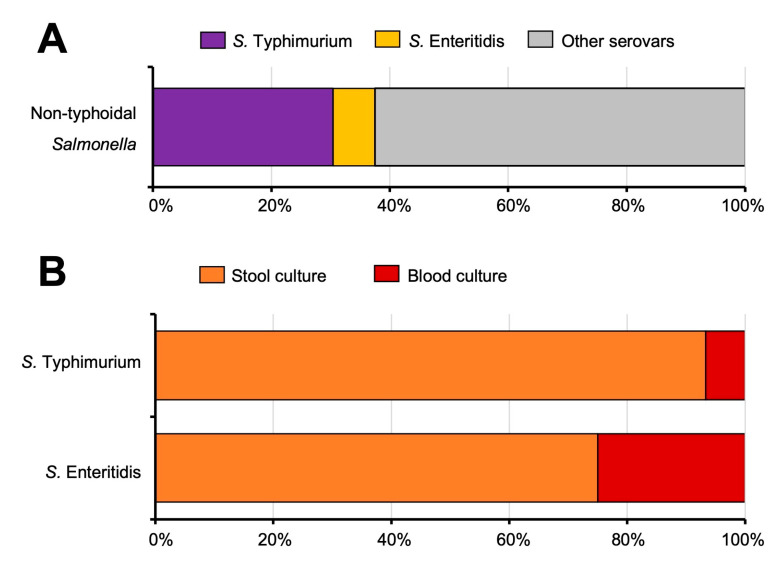
Percentage of *Salmonella enterica* serovar Typhimurium (*S. Typhimurium*), Enteritidis (*S. Enteritidis*), and other serovars (**A**), isolated from stool or blood culture (**B**) of hospitalized patients at Maharaj Nakorn Chiang Mai hospital from 2016 to 2021 (Total N = 395, 120 for *S. Typhimurium*, 28 for *S. Enteritidis* and 247 for other serovars).

From 2016 to 2021, a total of 395 NTS clinical isolates were obtained from the Central Diagnostic Microbiology Laboratory of MNCMH. Serotyping of the isolates by a multiplex PCR revealed that 120 isolates (30.4%) were identified as serovar Typhimurium and 28 isolates (7.1%) as serovar Enteritidis, with 247 (62.5%) isolates being part of neither *S. Typhimurium* nor *S. Enteritidis*. However, they were not identified in this study (Figure 1A). Most *S. Typhimurium* and *S. Enteritidis* isolates were obtained from stool culture, 93.3% (112/120) and 75.0% (21/28), respectively. 6.7% (8/120) of *S. Typhimurium* and 25.0% (7/28) of *S. Enteritidis* isolates were obtained from blood culture, indicating the incidence of iNTS in MNCMH (Figure 1B). Data of each isolate (N = 148) is demonstrated in Supplementary Table S2. The demographic and principal diagnosis of iNTS cases in this study is shown in Table 2.

### 3.2. Patients with Anemia Have a High Risk for Salmonella Typhimurium Infection

Next, we compared the demographic (age and gender) along with clinical and laboratory findings (sepsis, hematocrit, leukocyte, neutrophil, and lymphocyte counts) of 148 patients infected with either *S. Typhimurium* (n = 120) or *S. Enteritidis* (n = 28) as shown in Table 3. However, the complete blood count (CBC) data of some patients was unavailable. Our data showed that the mean age and gender were not significantly different between patients infected with *S. Typhimurium* and *S. Enteritidis*. Most patients with *S. Typhimurium* infection were 1 to 4-year-old children (41/120, 35.0%) followed by 19–59-year-old adults (39/120, 33.3%). Most *S. Enteritidis*-infected patients were adults (19–59 years old) or elderly (60–93 years old), with 46.4% (13/28) and 25.0% (7/28), respectively. The available CBC data indicated no differences in the presence of sepsis, the numbers of leukocyte, neutrophil, and lymphocyte counts between *S. Typhimurium* and *S. Enteritidis*-infected groups. Interestingly, there was a significant difference in the percentage of Hct level between the STM-infected (69/80, 86.3%) and the SE-infected group (10/18, 55.6%) (*p* = 0.006). By using a univariate analysis, it was found that patients with anemia (Hct level less than 13%) were at a higher risk for STM than SE infection (odd ratio of 5.018 with 95% confidence interval of 1.63–15.48).

### 3.3. Multidrug Resistance and ESBL-Producing Salmonella Strains Found in the Clinical Isolates

**Figure 2:**
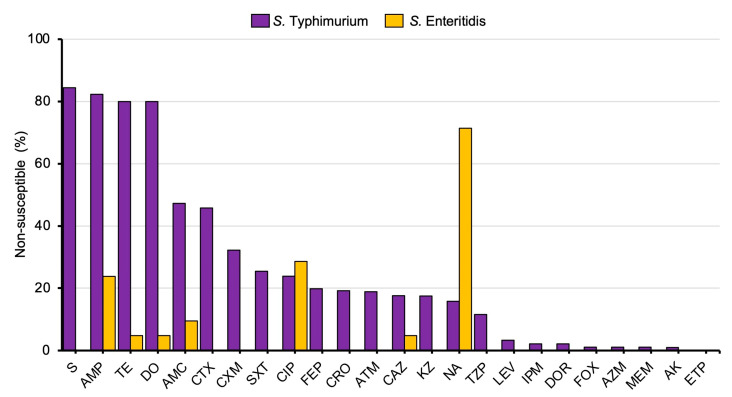
Distribution of *S. Typhimurium* and *S. Enteritidis* isolates that are non-susceptible to different types of antibacterial agents. (S, streptomycin; AMP, ampicillin; TE, tetracycline; DO, doxycycline; AMC, amoxicillin/clavulanic acid; CTX, cefotaxime; CXM, cefuroxime; SXT, trimethoprim/sulfamethoxazole; CIP, ciprofloxacin; FEP, cefepime; CRO, ceftriaxone; ATM, aztreonam; CAZ, ceftazidime; KZ, cephazolin; NA, nalidixic acid; TZP, piperacillin/tazobactam; LEV, levofloxacin; IPM, imipenem; DOR, doripenem; FOX, cefoxitin; AZM, azithromycin; MEM, meropenem; AK, amikacin; ETP, ertapenem).

**Figure 3:**
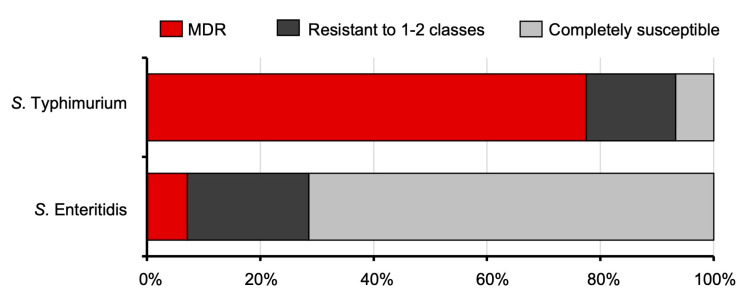
High multidrug resistance phenotype in *S. Typhimurium*. Percentage of the drug resistance phenotype (MDR, resistant to 1–2 classes and completely susceptible) of *S. Typhimurium* and *S. Enteritidis* clinically isolates. The MDR was defined as non-susceptible to at least one antimicrobial agent in ≥3 classes (Total N = 120 for STM, N = 28 for SE).

The *S. Typhimurium* and *S. Enteritidis* isolates were then tested for their susceptibility to 13 antimicrobial classes with a total of 24 antibacterial agents by a disc diffusion assay. The percentage of non-susceptible isolates between *S. Typhimurium* and *S. Enteritidis* is shown in Figure 2. Our data show that the highest incidence of non-susceptible antimicrobial agents is streptomycin (S), with 84.4% in *S. Typhimurium* but not *S. Enteritidis*. Among *S. Typhimurium* isolates, 82.3% are non-susceptible to ampicillin (AMP), followed by tetracycline (TE), doxycycline (DO), and amoxicillin-clavulanic acid (AMC) at 80.0%, 80.0%, and 47.3% of the isolates tested, respectively. Interestingly, *S. Enteritidis* isolates exhibited the highest non-susceptibility to nalidixic acid (NA), with 71.4%, followed by ciprofloxacin (CIP) and ampicillin (AMP), with 28.6% and 23.8%, respectively. Moreover, 6 out of 120 (5%) *S. Typhimurium* isolates, but none of the *S. Enteritidis* isolates produced extended-spectrum beta-lactamase (ESBL) detected by the double-disc synergy test (DDST). The source, antibiotic resistance pattern, MAR index, and virulence gene profiles of these 6 ESBL-positive *S. Typhimurium* isolates were shown in Supplementary Table S3. All *S. Typhimurium* isolates (120/120, 100%) are susceptible to ertapenem (ETP). All *S. Enteritidis* isolates are susceptible to most antibacterial agents tested except ampicillin (AMP), tetracycline (TE), doxycycline (DO), amoxicillin-clavulanic acid (AMC), ciprofloxacin (CIP), ceftazidime (CAZ), and nalidixic acid (NA). These data indicate that *S. Typhimurium* isolates in this study are more resistant to commonly used antibacterial agents than *S. Enteritidis*. *S. Typhimurium* conferred MDR phenotype higher than *S. Enteritidis* with multiple antibiotic resistance (MAR) indexes of 0.22 and 0.04 for *S. Typhimurium* and *S. Enteritidis*, respectively (Supplementary Tables S4 and S5). The percentage of *S. Typhimurium* isolates with a MDR phenotype (93/120, 77.5%) is significantly higher than that of *S. Enteritidis* (2/28, 7.1%) (Figure 3). Two *S. Typhimurium* isolates showed resistance across 10 of the 13 antibacterial agents tested, whereas the maximum resistance for *S. Enteritidis* was 4 classes, observed in only one isolate. 

### 3.4. Detection of Essential Salmonella Virulence Genes in Clinically Isolated Strains

**Figure 4:**
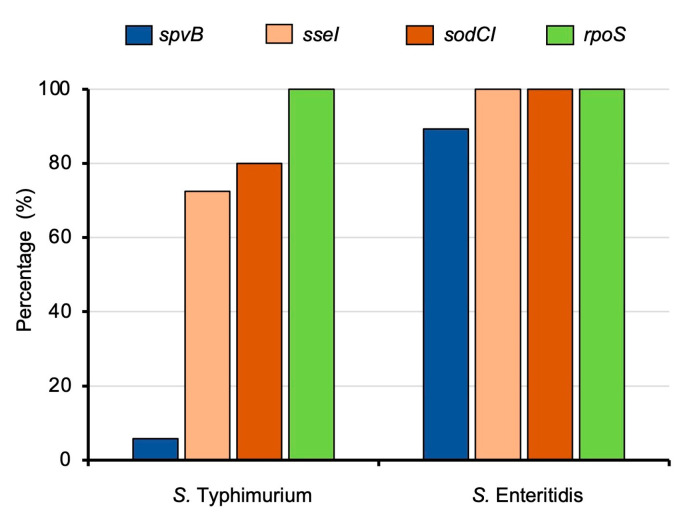
Percentage of the presence of important *Salmonella* virulence genes (*spvB*, *sseI*, *sodCI*, and *rpoS*) in *S. Typhimurium* and *S. Enteritidis* clinical isolates. (Total N = 120 for *S. Typhimurium*, N = 28 for *S. Enteritidis*).

**Figure 5:**
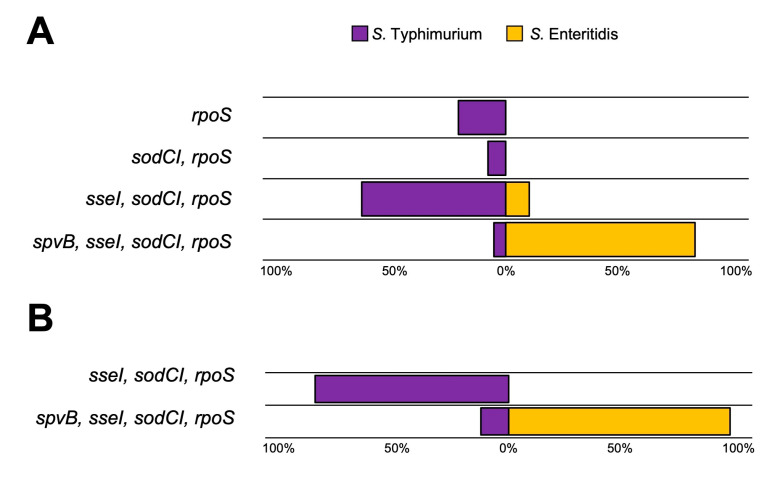
Virulence gene-harboring pattern of *S. Typhimurium* and *S. Enteritidis* clinically strains isolated from stool culture (**A**) and blood culture (**B**). (Total N = 120 for *S. Typhimurium*, N = 28 for *S. Enteritidis*).

Next, we detected the presence of four representative genes essential for *Salmonella* pathogenesis in a mammalian host (*spvB*, *sseI*, *sodCI*, and *rpoS*) by PCR using the primer pairs in Table 1. The distribution of these four virulence genes across all 148 clinical *Salmonella* isolates is shown in Figure 4. All clinical isolates of *S. Typhimurium* and *S. Enteritidis* harbor *rpoS* gene. The *sodCI* and *sseI* genes were detected in all *S. Enteritidis* isolates. However, not all strains of *S. Typhimurium* contain *sodCI* and *sseI* genes (80.0% and 72.5%, respectively). Interestingly, the presence of *spvB* gene in *S. Enteritidis* is significantly higher (89.3%) than that of *S. Typhimurium* (5.8%). The distribution pattern of these four *Salmonella* virulence genes for all the strains is shown in Figure 5. Most *S. Typhimurium* isolates (65.2%) harbor *sseI-sodCI-rpoS* genes pattern, while most SE isolates (89.3%) have *spvB-sseI-sodCI-rpoS* genes pattern. The same pattern of virulence genes was observed in stool and blood-isolated *S. Typhimurium* and *S. Enteritidis* strains (Figure 5A,B, respectively). All blood-isolated strains also have either *sseI-sodCI-rpoS* or *spvB-sseI-sodCI-rpoS* gene patterns.

### 3.5. MLST Revealed the Presence of MDR Salmonella Typhimurium ST34, ST19, and Salmonella Enteritidis ST11 

Finally, we randomly picked 20 out of 120 and 10 out of 28 strains of *S. Typhimurium* and *S. Enteritidis*, respectively, for sequence typing. The ST was assigned by MLST data based on an allelic profile of seven housekeeping genes (*aroC*, *dnaN*, *hemD*, *hisD*, *purE*, *sucA*, and *thrA*). Almost all the STM isolates (19/20, 95.0%) were ST34, except for one isolate (1/20, 5%) was ST19. All SE isolates (10/10, 100%) are ST11 (Table 4). The numbers and patterns of antimicrobial resistance of these isolates are shown in Table 5. Among these 30 MLST-typed isolates (N = 20 for ST34 *S. Typhimurium* and N = 10 for ST11 *S. Enteritidis*), 18/20 (90%) of ST34 *S. Typhimurium*, and 2/10 (20%) of ST11 *S. Enteritidis* have MDR phenotype. 2/18 (11.1%) of ST34 *S. Typhimurium* are resistant to 14 antimicrobial agents. The most frequent MDR pattern in ST34 *S. Typhimurium* is S-AMP-TE-DO (15/18, 83.3%). 

## 4. Discussion

Acute NTS is an important zoonotic foodborne illness that impacts human and animal health. Several livestock, such as pigs and poultry, are major reservoirs of drug-resistant nontyphoidal *Salmonella* [44]. Close contact between people and animals is usually found in some areas of Thailand and is considered an important epidemiological risk factor. Previous epidemiological reports confirmed the rise of MDR NTS isolates globally and regionally [3,6]. In this study, we focused on serovar Typhimurium and Enteritidis, the major human foodborne pathogens, and their increased MDR phenotype are of concern [45]. We collected a total of 395 isolates of *Salmonella* spp. from stool or blood culture positive for *Salmonella* spp. from hospitalized patients at MNCMH from 2016 to 2021. 

From the epidemiological studies of NTS in Thailand from 2002 to 2018, *S. Typhimurium* and *S. Enteritidis* were the major serovars detected from patient specimens, including stool and blood samples [46,47,48,49]. However, variation between the geographical locations was observed. For example, 19.1% of *S. Typhimurium* and 19.7% of *S. Enteritidis* were detected from NTS patients in Northern Thailand from 2002–2007 (a total of 11,656 isolates) [10,43]. In the large-scale study of Hendriksen et al., the authors reported that the top 10 serovars of nontyphoidal *Salmonella* in Northern Thailand are Enteritidis, Stanley, Weltevreden, Rissen, I (1),4,(5),12:i-, Choleraesuis, Anatum, Typhimurium, Corvallis, and Panama. Our study (N = 395) found that 30.4% are *S. Typhimurium*, 7.1% are *S. Enteritidis*, and 62.5% are other serovars. Most of *S. Typhimurium* (93.3%) and 75% of *S. Enteritidis* were isolated from the patient’s stool, while 6.7% of *S. Typhimurium* and 25.0% of *S. Enteritidis* were collected from the patient’s blood. These blood-isolated strains indicated the presence of iNTS at MNCMH during 2016–2021.

The demographic and principal diagnosis of 15 iNTS patients collected in our study (10.2%, 15 out of 148) is shown in Table 2. The age of iNTS patients can vary greatly from 2 to 76 years. Moreover, the immune status and Hct levels of patients were reported. Our data revealed no significant difference in age, gender, sepsis, leukocyte count, neutrophil count, and lymphocyte count between *S. Typhimurium* and *S. Enteritidis*-infected patients admitted at MNCMH. Interestingly, *S. Typhimurium* strains were significantly isolated more from anemic patients (86.3%), who have Hct levels less than 13%, than non-anemic patients (13.8%). Our study found that 80.6% of *S. Typhimurium* or *S. Enteritidis*-infected patients were anemic. This is consistent with the previous study showing that anemic patients are more susceptible to NTS [10]. Katz et al. found that the hemoglobin or Hct level is inversely related to NTS bacteremia in adults in a high-income country. An increase in hemoglobin level by 1 g/dL is associated with a 28% lower incidence of iNTS. Low hemoglobin levels might reflect the poor nutritional status of patients. To our knowledge, no direct evidence indicates that *S. Typhimurium* infection causes anemia in NTS. However, anemia could be a significant risk factor for a subsequent *S. Typhimurium* infection. 

In our study, *S. Enteritidis* can be isolated from stool and blood cultures, in accordance with the previous reports [50,51]. *S. Enteritidis* isolated in Thailand was associated with life-threatening bacteremia in an immunocompromised host. An observational study in 2009 showed that 60.8% of *S. Enteritidis* isolates (n = 1517) were recovered from blood specimens [51]. However, our study found that both *S. Typhimurium* and *S. Enteritidis* can cause NTS bacteremia. Our study also showed that *S. Typhimurium* isolates have a higher percentage of non-susceptibility to several antibacterial agents than *S. Enteritidis* isolates. *S. Typhimurium* isolates exhibited the highest resistance to streptomycin, followed by ampicillin, tetracycline, and doxycycline. The most frequent MDR pattern in ST34 *S. Typhimurium* in our study is streptomycin (S)-ampicillin (AMP) -TE (tetracycline)- doxycycline (DO) (15/18, 83.3%). Our results are consistent with the MDR phenotype of *S. Typhimurium* isolated from humans, pigs, pork, and poultry in previous reports [47,52]. *S. Enteritidis* clinical isolates exhibited the highest resistance to nalidixic acid followed by ciprofloxacin and ampicillin, which is consistent with the previous report [51]. Hendriksen et al. reported that *S. Enteritidis* isolates from blood and stool specimens in Thailand are resistant to ampicillin, ciprofloxacin, and nalidixic acid. A spread of the MDR ST11 *S. Enteritidis* between chickens and humans in Thailand had also been reported [52]. We found that some *S. Typhimurium* isolates (5%, 6/120) were ESBL-producers, but none of the *S. Enteritidis*. The previous study showed that ESBL-producing *Salmonella* (1.9%) were detected from slaughterhouse pigs and retail market pork in the border of Thailand [11].

The first to fourth-generation cephalosporins susceptibility of the *Salmonella* isolates were investigated in this study. A total of 21 *S. Typhimurium* isolates (21/120, 17.5%) resisted the second and third-generation cephalosporins, thus defined as extended-spectrum-cephalosporins (ESC) resistance strains. Moreover, 15% (18/120) of *S. Typhimurium* isolates were resisted to a fourth-generation cephalosporin, cefepime. The previous work illustrated that 3.8% of NTS isolated from pigs and pork in Thailand are resistant to a third-generation cephalosporin (ceftazidime, cefotaxime, or cefpodoxime) [11]. The cefepime-resistance *Salmonella* strains were also found in Phnom Penh, Cambodia, Taiwan, and Egypt [44,53,54]. The cefotaxime-resistance *Salmonella* could be a global threat since it was found in Finland by travelers returning from Thailand between 1993–2011 [55]. Our study also reported the presence of ESC and cefepime-resistance *S. Typhimurium* in Thailand.

Most *S. Typhimurium* confers *sseI*, *sodCI*, *rpoS* virulence gene patterns while most *S. Enteritidis* harbors *spvB*, *sseI*, *sodCI*, *rpoS*. The *sseI* and *spvB* genes can be detected from both stool and blood isolates (*sseI* for *S. Typhimurium*, *spvB* and *sseI* for *S. Enteritidis*). This data indicated the role of *Salmonella sseI* and *spvB* genes in human systemic infection. *Salmonella* plasmid virulence (*spv*) genes located on the pSTV plasmid contribute to *Salmonella* systemic dissemination and are found only in eight serovars, including Typhimurium and Enteritidis [56]. In our study, most *S. Enteritidis* isolates contain the *spvB* gene but only a minor portion of *S. Typhimurium* contains the *spvB* gene. This finding is consistent with our previous report showing that *S. Typhimurium* ST34 isolated from Chiang Mai, Thailand, lacked *spvB* gene and resulted in attenuated pathogenicity in mice [35]. Majority of *Salmonella* isolates in this study have *sodCI* gene in which encodes a superoxide dismutase essential for *Salmonella* systemic dissemination and bacteremia in mammalian host [57]. All *S. Typhimurium* and *S. Enteritidis* isolates in this study contain the *rpoS* gene, encoding for the RNA polymerase sigma factor (RpoS). The RpoS plays a critical role in *Salmonella* survival under stress conditions and regulates the expression of *spv* locus. Our data of *rpoS* distribution is consistent with previous reports of human NTS [29,58]. 

Most *S. Typhimurium* and *S. Enteritidis* clinical isolates in this study belong to ST34 and ST11, respectively, with one isolate is *S. Typhimurium* ST19. The monophasic ST34 and ST19 *S. Typhimurium* have been reported globally for their outbreak in food and animals [24,26,59,60,61,62]. The recent work illustrated a rapid multiplex PCR method to detect the monophasic variant of S. Typhimurium [63]. Nevertheless, the presence of monophasic Typhimurium, a significant increase in human salmonellosis, was not included in our study. *S. Enteritidis* ST11 is among Southeast Asia’s most frequent STs associated with NTS bacteremia [25,64]. 

## 5. Conclusions

We reported that (i) *S. enterica* Typhimurium and Enteritidis are among the frequent serovars of NTS isolated from stool and blood of humans in Chiang Mai, Thailand, between 2016–2021 (ii) Anemic patients are at high risk for *S. Typhimurium* infection (iii) Higher prevalence of MDR *S. Typhimurium* compared to that of *S. Enteritidis* (iv) Most *S. Enteritidis* ST11 contain virulence gene *sseI*, *sodCI*, *rpoS* and *spvB*, while most *S. Typhimurium* ST34 lack *spvB.* However, the relevance of our study is still limited by its comparatively low sample size, especially in the ST identification. Further investigations on *S. Typhimurium* and *S. Enteritidis* genomics, especially the presence of antimicrobial resistance genes and their transmission, would benefit the strategies for preventing MDR *S. Typhimurium* and *S. Enteritidis* from spreading in the community.

## Figures and Tables

**Table 1:** Primer pairs used in this study.

	Target Gene (Synonym)	Sequence	Reference
*Salmonella* spp.	STM3098	5’-TTTGGCGGCGCAGGCGATTC-3’ 5’-GCCTCCGCCTCATCAATCCG-3’	[36]
*S. Typhimurium* (STM)	STM4497M2	5’-AACAACGGCTCCGGTAATGAGATTG-3’ 5’-ATGACAAACTCTTGATTCTGAAGATCG-3’	
*S. Enteritidis* (SE)		5’-GGATAAGGGATCGATAATTGCTCAC-3’ 5’-GGACTTCCAGTTATAGTAGGTGGCC-3’	
Important virulence genes	*spvB*	5’-GACTATCTTTCCACAAATGAACCC-3’ 5’-GTATCTATGAGTTGAGTACCTC-3’	[29]
	*sseI*	5’-TCCGCCGATAACCTTATTGTG-3’ 5’-CTGTCATCTGTGATAGTGTCC-3’	
	*sodCI*	5’-TATCGGAGTAATTGTCACCG-3’ 5’-ACAATATTGTCGCTGGTAGC-3’	
	*rpoS*	5’-TGCTGGCAGAAGACAAACGG-3’ 5’-TGATTACCTGAGTGCCTACG-3’	
MLST	*aroC*	5’-CCTGGCACCTCGCGCTATAC-3’ 5’-CCACACACGGATCGTGGCG-3’	[37]
	*dnaN*	5’-ATGAAATTTACCGTTGAACGTGA-3’ 5’-AATTTCTCATTCGAGAGGATTGC-3’	
	*hemD*	5’-ATGAGTATTCTGATCACCCG-3’ 5’-ATCAGCGACCTTAATATCTTGCCA-3’	
	*hisD*	5’-GAAACGTTCCATTCCGCGCAGAC-3’ 5’-CTGAACGGTCATCCGTTTCTG-3’	
	*purE*	5’-ATGTCTTCCCGCAATAATCC-3’ 5’-TCATAGCGTCCCCCGCGGATC-3’	
	*sucA*	5’-AGCACCGAAGAGAAACGCTG-3’ 5’-GGTTGTTGATAACGATACGTAC-3’	
	*thrA*	5’-GTCACGGTGATCGATCCGGT-3’ 5’-CACGATATTGATATTAGCCCG-3’	

**Table 2:** Demographic and principal diagnosis of iNTS patients (15 out of 148 patients).

Age (Year)	Sex	Principal Diagnosis	Hct Level (mg/dL) on Admission Date
68	M	Tuberculous spondylitis	31.6
8	M	*Salmonella* septicemia	22.6
60	M	Acute myeloid leukemia	19.8
24	M	Human immunodeficiency virus (HIV) infection	32.1
43	M	HIV infection	19.9
2	F	Acute lymphoblastic leukemia	29.1
76	F	*Salmonella* septicemia	23.3
43	F	Secondary malignant neoplasm of brain	36.8
71	M	*Salmonella* septicemia	not available
56	F	Intracranial brain abscess (post-surgery)	42.1
58	M	Pericarditis	29.5
23	M	Human immunodeficiency virus (HIV) infection	39.9
59	F	Acute febrile illness with pneumonia	35.8
64	F	Acute febrile illness	30.5
52	F	Adult-onset immunodeficiency disease	31.4

M, male; F, female; Hct, hematocrit.

**Table 3:** Demographic and clinical data of hospitalized patients with stool or blood culture positive for *Salmonella enterica* serovars.

Characteristic	Total n (%)	*S. Typhimurium* n (%)	*S. Enteritidis* n (%)	*p*-Value
Mean age (SD), year	29.3 (27.9)	27.8 (28.2)	35.4 (26.3)	0.466
Age (year)				0.275
<1	9 (6.2)	9 (7.7)	0	
1–4	47 (32.4)	41 (35.0)	6 (21.4)	
5–18	8 (5.5)	6 (5.1)	2 (7.1)	
19–59	52 (35.9)	39 (33.3)	13 (46.4)	
60–93	29 (20.0)	22 (18.8)	7 (25.0)	
Gender				0.284
Male	81 (55.4)	68 (57.6)	15 (53.6)	
Female	65 (44.5)	50 (42.4)	13 (46.4)	
Sepsis				0.570
Sepsis	49 (50.5)	39 (48.1)	10 (55.6)	
Non-sepsis	43 (49.5)	35 (51.9)	8 (44.4)	
Hematocrit (Hct) (%)				0.006 **
Decreased (<13)	79 (80.6)	69 (86.3)	10 (55.6)	
Normal (13–18)	19 (19.4)	11 (13.8)	8 (44.4)	
Leukocyte (cells/mm^3^)				0.805
Increased (>10,000)	34 (34.7)	28 (35.0)	6 (33.3)	
Decreased (<5000)	21 (21.4)	18 (22.5)	3 (16.7)	
Normal (5000–10,000)	43 (43.9)	34 (42.5)	9 (50.0)	
Neutrophil (cells/mm^3^)				0.386
Increased (>74)	42 (42.9)	32 (40.0)	10 (55.6)	
Decreased (<40)	9 (9.2)	7 (8.8)	2 (11.1)	
Normal (40–74)	47 (48.0)	41 (51.2)	6 (33.3)	
Lymphocyte (cells/mm^3^)				0.507
Increased (>48)	9 (9.2)	8 (10.0)	1 (5.6)	
Decreased (<19)	48 (49.0)	37 (46.3)	11 (61.1)	
Normal (19–48)	41 (41.8)	35 (43.8)	6 (33.3)	

** *p* < 0.01.

**Table 4:** MLST pattern of 30 clinical *Salmonella enterica* serovars.

Sequence Type (ST)	No. of Isolates	Allelic Profile						
		*aroC*	*dnaN*	*hemD*	*hisD*	*purE*	*sucA*	*thrA*
***S. Typhimurium***								
ST34	19	10	19	12	9	5	9	2
ST19	1	10	7	12	9	5	9	2
***S. Enteritidis***								
ST11	10	5	2	3	7	6	6	11

**Table 5:** The emergence of MDR *Salmonella enterica* Typhimuirium ST34 and *Salmonella enterica* Enteritidis ST11.

ST	Sample ID	Year	Source	No. of Agents	Antimicrobial Resistance Patterns
***S. Typhimurium***					
ST34	CNTS005	2016	Blood	13	S-AMP-TE-DO-CTX-CXM-CIP-FEP-CRO-ATM-CAZ-KZ-NA
ST34	CNTS012	2016	Stool	4	S-AMP-TE-DO
ST34	CNTS019	2016	Stool	14	S-AMP-TE-DO-CTX-CXM-SXT-FEP-CRO-ATM-CAZ-KZ-NA-TZP
ST34	CNTS023	2016	Stool	5	S-AMP-TE-DO-CXM
ST34	CNTS040	2017	Stool	5	S-AMP-TE-DO-CXM
ST34	CNTS041	2017	Stool	5	S-AMP-TE-DO-AMC
ST34	CNTS046	2017	Stool	4	S-AMP-TE-DO
ST34	CNTS056	2017	Blood	4	S-TE-DO-SXT
ST34	CNTS065	2017	Stool	5	S-AMP-TE-DO-AMC
ST34	CNTS069	2017	Stool	5	S-AMP-TE-DO-AMC
ST34	CNTS071	2017	Stool	5	S-AMP-TE-DO-AZM
ST34	CNTS075	2017	Stool	6	S-AMP-TE-DO-SXT-CIP
ST34	CNTS080	2017	Stool	7	S-AMP-TE-DO-AMC-SXT-CIP
ST34	CNTS089	2017	Stool	11	S-AMP-TE-DO-CTX-CXM-FEP-CRO-ATM-CAZ-KZ
ST34	CNTS091	2017	Stool	10	S-TE-DO-CTX-CXM-FEP-CRO-ATM-CAZ-KZ
ST34	CNTS103	2017	Stool	14	S-AMP-TE-DO-AMC-CTX-CXM-SXT-CIP-FEP-CRO-ATM-CAZ-KZ
ST34	CNTS119	2018	Stool	7	S-AMP-TE-DO-CTX-CXM-NA
ST34	CNTS147	2018	Stool	3	S-AMP-CXM
***S. Enteritidis***					
ST11	CNTS109	2018	Stool	4	AMP-AMC-CIP-NA
ST11	CNTS112	2018	Stool	6	AMP-TE-DO-AMC-CIP-NA

S, Streptomycin; AMP, Ampicillin; TE, Tetracycline; DO, Doxycycline; AMC, Amoxicillin/clavulanic acid; CTX, Cefotaxime; CXM, Cefuroxime; SXT, Trimethoprim/Sulfamethoxazole; CIP, Ciprofloxacin; FEP, Cefepime; CRO, Ceftriaxone; ATM, Aztreonam; CAZ, Ceftazidime; KZ, Cephazolin; NA, Nalidixic acid; TZP, Piperacillin/Tazobactam.

## Data Availability

The sequences for Salmonella seven housekeeping genes (*aroC*, *dnaN*, *hemD*, *sucA*, *thrA*, *purE*, and *hisD*) of the isolates in this study were deposited to the GenBank database under the accession numbers OR504765 to OR504974. Please contact the corresponding author for other data requests.

## Supplementary Materials

The following supporting information can be downloaded at: https://www.mdpi.com/article/10.3390/microorganisms11102425/s1, Table S1: Classification of 24 antimicrobial agents in 13 antimicrobial classes and their abbreviations used in this study.; Table S2: Demographic and clinical data of hospitalized patients with stool or blood culture positive for *Salmonella enterica* Serovar Typhimurium and Enteritidis.; Table S3: The source, antibiotic resistance pattern, multiple antibiotic resistance (MAR) index, and virulence gene profiles of 6 ESBL-positive *S. Typhimurium* isolates.; Table S4: The multiple antibiotic resistance (MAR) index of each *S. Typhimurium* isolate (N = 120).; Table S5: The multiple antibiotic resistance (MAR) index of each *S. Enteritidis* isolate (N = 28).
