# Supplementary material for: Clinical Characteristics, Antimicrobial Resistance, Virulence Genes and Multi-Locus Sequence Typing of Non-Typhoidal Salmonella Serovar Typhimurium and Enteritidis Strains Isolated from Patients in Chiang Mai, Thailand
Source: Microorganisms. 2023 Sep 28;11(10):2425. doi: 10.3390/microorganisms11102425 (PMC10609586; doi:10.3390/microorganisms11102425)
Supplement: Supplementary file 1 [file microorganisms-11-02425-s001.zip › microorganisms-2570571-supplementary.pdf]

Supplementary Table 1: Classification of 24 antimicrobial agents in 13 antimicrobial classes and their abbreviations used in this study

Antimicrobial class	Antimicrobial agent
Aminoglycosides	Streptomycin (S), amikacin (AK)
Antipseudomonal penicillins with β-lactamase inhibitors	Piperacillin/tazobactam (TZP)
Carbapenems	Imipenem (IPM), doripenem (DOR), meropenem (MEM)
Non-extended spectrum cephalosporins; 1st and 2nd generation cephalosporins	Cefuroxime (CXM), ceftriaxone (CRO), cephazolin (KZ), ertapenem (ETP)
Extended-spectrum cephalosporins; 3rd and 4th generation cephalosporins	Cefotaxime (CTX), cefepime (FEP), ceftazidime (CAZ)
Cephamycins	Cefoxitin (FOX)
Fluoroquinolones	Ciprofloxacin (CIP), nalidixic acid (NA), levofloxacin (LEV)
Folate pathway inhibitors	Trimethoprim/sulfamethoxazole (SXT)
Monobactams	Aztreonam (ATM)
Penicillins	Ampicillin (AMP)
Penicillins with β-lactamase inhibitors	Amoxicillin/clavulanic acid (AMC)
Tetracyclines	Tetracycline (TE), doxycycline (DO)
Macrolides	Azithromycin (AZM)

Supplementary Table 2: Demographic and clinical data of hospitalized patients with stool or blood culture positive for *Salmonella enterica* Serovar Typhimurium and Enteritidis (1/8).

Sample ID	Year	Source	Serovar	Age (Year)	Gender	Sepsis	Hct (%)	Leuk. (cells/mm^3)	Neutro. (cells/mm^3)	Lympho. (cells/mm^3)	*spvB*	*sseI*	*sodCI*	*rpoS*
CNTS005	2016	blood	STM	58	M	Yes	normal	increased	increased	decreased	-	+	+	+
CNTS009	2016	stool	SE	66	F	Yes	normal	increased	increased	decreased	+	+	+	+
CNTS010	2016	blood	SE	68	M	Yes	increased	increased	decreased	decreased	+	+	+	+
CNTS012	2016	stool	STM	66	F	Yes	normal	increased	increased	decreased	-	+	+	+
CNTS016	2016	stool	STM	0.75	F	N/A	N/A	N/A	N/A	N/A	-	+	+	+
CNTS017	2016	stool	STM	1	M	N/A	N/A	N/A	N/A	N/A	-	+	+	+
CNTS019	2016	stool	STM	1	M	N/A	N/A	N/A	N/A	N/A	-	+	+	+
CNTS020	2016	stool	SE	1	M	N/A	N/A	N/A	N/A	N/A	+	+	+	+
CNTS023	2016	stool	STM	0.67	F	N/A	N/A	N/A	N/A	N/A	-	+	+	+
CNTS024	2016	blood	SE	23	M	Yes	decreased	decreased	increased	decreased	+	+	+	+
CNTS028	2016	blood	STM	8	M	Yes	decreased	increased	increased	decreased	-	+	+	+
CNTS029	2016	stool	STM	1	F	N/A	N/A	N/A	N/A	N/A	-	+	+	+
CNTS030	2016	blood	SE	60	M	Yes	decreased	decreased	decreased	increased	+	+	+	+
CNTS031	2016	stool	STM	1	M	No	decreased	decreased	normal	normal	-	+	+	+
CNTS032	2016	stool	STM	23	M	N/A	N/A	N/A	N/A	N/A	-	+	+	+
CNTS037	2016	stool	STM	18	F	Yes	decreased	increased	increased	decreased	+	+	+	+
CNTS039	2016	blood	STM	71	F	N/A	N/A	N/A	N/A	N/A	-	+	+	+
CNTS040	2017	stool	STM	0	F	N/A	N/A	N/A	N/A	N/A	-	+	+	+
CNTS041	2017	stool	STM	1	M	Yes	decreased	increased	normal	normal	-	+	+	+
CNTS045	2017	stool	STM	9	F	Yes	decreased	increased	increased	decreased	+	+	+	+

STM, *Salmonella enterica* Typhimurium; SE, *S. enterica* Enteritidis; M, male; F, female; N/A, Data not available; Hct, hematocrit (normal = 13-18%); Leuk, leukocyte (normal = 5000 – 10000 cells/mm^3); Neutro, neutrophil (normal = 40-74 cells/mm^3) and Lympho, lymphocyte (normal = 19-48 cells/mm^3); +, presence; -, absence

Supplementary Table 2: Demographic and clinical data of hospitalized patients with stool or blood culture positive for *Salmonella enterica* Serovar Typhimurium and Enteritidis (2/8).

Sample ID	Year	Source	Serovar	Age (Year)	Gender	Sepsis	Hct (%)	Leuk. (cells/mm^3)	Neutro. (cells/mm^3)	Lympho. (cells/mm^3)	*spvB*	*sseI*	*sodCI*	*rpoS*
CNTS046	2017	Stool	STM	1	M	Yes	normal	normal	normal	normal	-	+	+	+
CNTS047	2017	Stool	STM	1	F	Yes	decreased	increased	normal	normal	-	+	+	+
CNTS048	2017	Stool	STM	59	M	Yes	decreased	decreased	normal	normal	-	-	-	+
CNTS049	2017	Blood	STM	24	M	Yes	decreased	decreased	normal	normal	+	+	+	+
CNTS050	2017	Stool	STM	72	F	N/A	N/A	N/A	N/A	N/A	-	-	-	+
CNTS053	2017	Stool	STM	64	F	Yes	decreased	increased	increased	decreased	+	+	+	+
CNTS055	2017	Stool	STM	37	M	Yes	decreased	decreased	decreased	increased	-	-	-	+
CNTS056	2017	Blood	STM	43	M	No	decreased	normal	increased	decreased	-	+	+	+
CNTS058	2017	Stool	STM	2	F	Yes	normal	normal	increased	decreased	-	+	+	+
CNTS059	2017	Stool	SE	76	F	No	decreased	normal	increased	decreased	+	+	+	+
CNTS060	2017	Blood	STM	43	M	Yes	decreased	decreased	increased	decreased	-	+	+	+
CNTS064	2017	Stool	STM	1	F	N/A	N/A	N/A	N/A	N/A	-	-	-	+
CNTS065	2017	Stool	STM	64	F	Yes	decreased	normal	increased	decreased	-	+	+	+
CNTS068	2017	Stool	STM	54	M	No	normal	increased	increased	decreased	-	+	+	+
CNTS069	2017	Stool	STM	54	M	No	normal	normal	increased	decreased	-	+	+	+
CNTS070	2017	Stool	STM	1	M	Yes	decreased	normal	normal	normal	-	+	+	+
CNTS071	2017	Stool	STM	1	F	N/A	N/A	N/A	N/A	N/A	-	+	+	+
CNTS073	2017	Stool	STM	1	M	Yes	decreased	increased	increased	decreased	-	+	+	+
CNTS074	2017	Stool	SE	23	F	Yes	decreased	increased	increased	decreased	+	+	+	+
CNTS075	2017	Stool	STM	62	F	Yes	decreased	normal	increased	decreased	-	+	+	+

STM, *Salmonella enterica* Typhimurium; SE, *S. enterica* Enteritidis; M, male; F, female; N/A, Data not available; Hct, hematocrit (normal = 13-18%); Leuk, leukocyte (normal = 5000 – 10000 cells/mm^3); Neutro, neutrophil (normal = 40-74 cells/mm^3) and Lympho, lymphocyte (normal = 19-48 cells/mm^3); +, presence; -, absence

Supplementary Table 2: Demographic and clinical data of hospitalized patients with stool or blood culture positive for *Salmonella enterica* Serovar Typhimurium and Enteritidis (3/8).

Sample ID	Year	Source	Serovar	Age (Year)	Gender	Sepsis	Hct (%)	Leuk. (cells/mm^3)	Neutro. (cells/mm^3)	Lympho. (cells/mm^3)	*spvB*	*sseI*	*sodCI*	*rpoS*
CNTS078	2017	Stool	STM	1	M	N/A	N/A	N/A	N/A	N/A	-	+	+	+
CNTS080	2017	Stool	STM	0	F	No	decreased	normal	normal	normal	-	+	+	+
CNTS081	2017	Stool	STM	60	M	No	normal	normal	normal	normal	-	-	+	+
CNTS083	2017	Stool	STM	2	F	N/A	N/A	N/A	N/A	N/A	-	+	+	+
CNTS085	2017	Stool	STM	3	M	No	normal	normal	decreased	increased	-	-	-	+
CNTS086	2017	Stool	STM	41	F	N/A	N/A	N/A	N/A	N/A	-	+	+	+
CNTS089	2017	Stool	STM	1	M	N/A	N/A	N/A	N/A	N/A	-	+	+	+
CNTS090	2017	Blood	STM	2	F	No	decreased	decreased	decreased	increased	-	+	+	+
CNTS091	2017	Stool	STM	N/A	N/A	N/A	N/A	N/A	N/A	N/A	-	-	+	+
CNTS093	2017	Stool	STM	41	M	N/A	N/A	N/A	N/A	N/A	-	-	-	+
CNTS094	2017	Stool	STM	26	M	N/A	N/A	N/A	N/A	N/A	-	-	+	+
CNTS097	2017	Stool	STM	0	F	Yes	decreased	increased	increased	decreased	-	-	+	+
CNTS098	2017	Stool	STM	72	M	Yes	decreased	increased	normal	normal	-	-	+	+
CNTS099	2017	Stool	STM	69	M	No	decreased	normal	normal	decreased	-	-	-	+
CNTS100	2017	Stool	STM	4	F	No	N/A	N/A	N/A	N/A	-	+	+	+
CNTS101	2017	Stool	STM	82	F	Yes	decreased	normal	increased	decreased	-	-	-	+
CNTS103	2017	Stool	STM	72	M	Yes	decreased	increased	normal	normal	-	-	+	+
CNTS105	2017	Stool	SE	26	M	No	normal	normal	normal	normal	+	+	+	+
CNTS107	2017	Stool	STM	69	M	No	decreased	normal	normal	decreased	-	-	-	+
CNTS109	2018	Stool	SE	10	M	No	decreased	increased	increased	decreased	+	+	+	+

STM, *Salmonella enterica* Typhimurium; SE, *S. enterica* Enteritidis; M, male; F, female; N/A, Data not available; Hct, hematocrit (normal = 13-18%); Leuk, leukocyte (normal = 5000 – 10000 cells/mm^3); Neutro, neutrophil (normal = 40-74 cells/mm^3) and Lympho, lymphocyte (normal = 19-48 cells/mm^3); +, presence; -, absence

Supplementary Table 2: Demographic and clinical data of hospitalized patients with stool or blood culture positive for *Salmonella enterica* Serovar Typhimurium and Enteritidis (4/8).

Sample ID	Year	Source	Serovar	Age (Year)	Gender	Sepsis	Hct (%)	Leuk. (cells/mm^3)	Neutro. (cells/mm^3)	Lympho. (cells/mm^3)	*spvB*	*sseI*	*sodCI*	*rpoS*
CNTS112	2018	Stool	SE	41	F	No	decreased	normal	increased	decreased	+	+	+	+
CNTS113	2018	Stool	STM	57	F	N/A	N/A	N/A	N/A	N/A	-	-	-	+
CNTS114	2018	Stool	STM	2	M	Yes	decreased	increased	normal	normal	-	-	+	+
CNTS115	2018	Blood	SE	59	F	Yes	normal	normal	increased	decreased	+	+	+	+
CNTS118	2018	Stool	STM	N/A	N/A	N/A	N/A	N/A	N/A	N/A	-	+	+	+
CNTS119	2018	Stool	STM	2	F	No	decreased	decreased	decreased	increased	-	+	+	+
CNTS123	2018	Stool	STM	1	M	N/A	N/A	N/A	N/A	N/A	-	+	+	+
CNTS125	2018	Stool	STM	38	M	Yes	decreased	decreased	normal	normal	-	-	+	+
CNTS126	2018	Stool	STM	35	F	Yes	decreased	normal	normal	normal	-	-	-	+
CNTS133	2018	Stool	STM	N/A	M	No	decreased	normal	normal	decreased	-	+	+	+
CNTS135	2018	Stool	STM	8	M	No	decreased	normal	increased	decreased	-	+	+	+
CNTS139	2018	Stool	STM	6	F	Yes	decreased	decreased	normal	decreased	-	+	+	+
CNTS141	2018	Stool	STM	30	M	N/A	N/A	N/A	N/A	N/A	-	-	-	+
CNTS144	2018	Stool	STM	66	F	Yes	decreased	increased	increased	decreased	-	-	+	+
CNTS147	2018	Stool	STM	1	M	Yes	normal	increased	increased	decreased	-	+	+	+
CNTS156	2018	Stool	STM	52	M	N/A	N/A	N/A	N/A	N/A	-	-	-	+
CNTS161	2018	Stool	SE	22	M	No	normal	normal	normal	normal	+	+	+	+
CNTS163	2018	Stool	STM	65	F	N/A	N/A	N/A	N/A	N/A	-	+	+	+
CNTS167	2018	Stool	STM	1	M	N/A	N/A	N/A	N/A	N/A	-	+	+	+
CNTS171	2018	Stool	STM	1	M	N/A	N/A	N/A	N/A	N/A	-	+	+	+

STM, *Salmonella enterica* Typhimurium; SE, *S. enterica* Enteritidis; M, male; F, female; N/A, Data not available; Hct, hematocrit (normal = 13-18%); Leuk, leukocyte (normal = 5000 – 10000 cells/mm^3); Neutro, neutrophil (normal = 40-74 cells/mm^3) and Lympho, lymphocyte (normal = 19-48 cells/mm^3); +, presence; -, absence

Supplementary Table 2: Demographic and clinical data of hospitalized patients with stool or blood culture positive for *Salmonella enterica* Serovar Typhimurium and Enteritidis (5/8).

Sample ID	Year	Source	Serovar	Age (Year)	Gender	Sepsis	Hct (%)	Leuk. (cells/mm^3)	Neutro. (cells/mm^3)	Lympho. (cells/mm^3)	*spvB*	*sseI*	*sodCI*	*rpoS*
CNTS172	2018	Stool	STM	23	M	No	decreased	normal	normal	decreased	-	+	+	+
CNTS174	2018	Stool	STM	1	M	N/A	N/A	N/A	N/A	N/A	-	+	+	+
CNTS177	2018	Stool	SE	19	M	No	normal	normal	increased	decreased	+	+	+	+
CNTS182	2018	Stool	SE	62	M	Yes	normal	decreased	normal	normal	+	+	+	+
CNTS183	2018	Stool	STM	93	F	No	decreased	normal	increased	decreased	-	+	+	+
CNTS185	2018	Stool	STM	79	M	No	decreased	normal	normal	normal	-	+	+	+
CNTS191	2018	Stool	SE	53	F	N/A	N/A	N/A	N/A	N/A	+	+	+	+
CNTS194	2018	Stool	SE	2	M	N/A	N/A	N/A	N/A	N/A	+	+	+	+
CNTS195	2018	Stool	STM	1	M	No	decreased	normal	normal	normal	-	+	+	+
CNTS199	2018	Stool	STM	2	M	No	decreased	normal	normal	normal	-	+	+	+
CNTS200	2018	Stool	SE	78	F	Yes	decreased	increased	increased	decreased	-	+	+	+
CNTS206	2018	Stool	STM	1	M	Yes	decreased	increased	decreased	increased	-	+	+	+
CNTS209	2018	Blood	STM	76	F	Yes	decreased	increased	increased	decreased	-	+	+	+
CNTS210	2018	Stool	SE	31	F	N/A	N/A	N/A	N/A	N/A	-	+	+	+
CNTS214	2018	Stool	STM	1	M	N/A	N/A	N/A	N/A	N/A	-	+	+	+
CNTS219	2018	Stool	STM	1	F	N/A	N/A	N/A	N/A	N/A	-	+	+	+
CNTS222	2018	Stool	STM	54	M	N/A	N/A	N/A	N/A	N/A	-	+	+	+
CNTS226	2018	Stool	STM	1	F	No	decreased	normal	normal	increased	-	-	-	+
CNTS227	2018	Stool	STM	24	F	No	normal	normal	normal	normal	-	+	+	+
CNTS230	2018	Stool	SE	42	F	N/A	N/A	N/A	N/A	N/A	-	+	+	+

STM, *Salmonella enterica* Typhimurium; SE, *S. enterica* Enteritidis; M, male; F, female; N/A, Data not available; Hct, hematocrit (normal = 13-18%); Leuk, leukocyte (normal = 5000 – 10000 cells/mm^3); Neutro, neutrophil (normal = 40-74 cells/mm^3) and Lympho, lymphocyte (normal = 19-48 cells/mm^3); +, presence; -, absence

Supplementary Table 2: Demographic and clinical data of hospitalized patients with stool or blood culture positive for *Salmonella enterica* Serovar Typhimurium and Enteritidis (6/8).

Sample ID	Year	Source	Serovar	Age (Year)	Gender	Sepsis	Hct (%)	Leuk. (cells/mm^3)	Neutro. (cells/mm^3)	Lympho. (cells/mm^3)	*spvB*	*sseI*	*sodCI*	*rpoS*
CNTS231	2018	Stool	STM	37	F	N/A	N/A	N/A	N/A	N/A	-	-	-	+
CNTS233	2018	Stool	STM	38	M	N/A	N/A	N/A	N/A	N/A	-	+	+	+
CNTS237	2018	Stool	STM	29	M	N/A	N/A	N/A	N/A	N/A	-	-	-	+
CNTS238	2018	Stool	STM	55	F	No	decreased	normal	increased	decreased	-	-	-	+
CNTS240	2018	Stool	STM	27	M	No	decreased	normal	normal	normal	-	-	-	+
CNTS246	2018	Stool	STM	65	M	No	decreased	normal	normal	decreased	-	+	+	+
CNTS251	2018	Stool	SE	2	F	N/A	N/A	N/A	N/A	N/A	+	+	+	+
CNTS252	2018	Stool	STM	1	M	No	decreased	normal	normal	normal	-	+	+	+
CNTS255	2019	Stool	STM	1	M	N/A	N/A	N/A	N/A	N/A	-	+	+	+
CNTS259	2019	Stool	STM	37	M	No	normal	normal	normal	normal	-	+	+	+
CNTS261	2019	Blood	SE	43	F	No	decreased	normal	normal	normal	+	+	+	+
CNTS270	2020	Stool	SE	4	M	N/A	N/A	N/A	N/A	N/A	+	+	+	+
CNTS272	2020	Stool	SE	1	F	N/A	N/A	N/A	N/A	N/A	+	+	+	+
CNTS277	2020	Stool	STM	1	F	No	decreased	increased	normal	normal	-	+	+	+
CNTS281	2020	Stool	STM	0.92	M	No	decreased	increased	normal	normal	-	+	+	+
CNTS282	2020	Stool	STM	0.75	M	No	decreased	increased	normal	normal	-	+	+	+
CNTS285	2020	Stool	STM	2	M	N/A	N/A	N/A	N/A	N/A	-	+	+	+
CNTS289	2020	Stool	STM	0.5	F	No	decreased	increased	normal	normal	-	+	+	+
CNTS290	2020	Stool	STM	34	F	No	decreased	normal	increased	decreased	-	+	+	+
CNTS291	2020	Stool	STM	56	F	Yes	decreased	increased	increased	decreased	+	+	+	+

STM, *Salmonella enterica* Typhimurium; SE, *S. enterica* Enteritidis; M, male; F, female; N/A, Data not available; Hct, hematocrit (normal = 13-18%); Leuk, leukocyte (normal = 5000 – 10000 cells/mm^3); Neutro, neutrophil (normal = 40-74 cells/mm^3) and Lympho, lymphocyte (normal = 19-48 cells/mm^3); +, presence; -, absence

Supplementary Table 2: Demographic and clinical data of hospitalized patients with stool or blood culture positive for *Salmonella enterica* Serovar Typhimurium and Enteritidis (7/8).

Sample ID	Year	Source	Serovar	Age (Year)	Gender	Sepsis	Hct (%)	Leuk. (cells/mm^3)	Neutro. (cells/mm^3)	Lympho. (cells/mm^3)	*spvB*	*sseI*	*sodCI*	*rpoS*
CNTS297	2020	Stool	SE	6	M	N/A	N/A	N/A	N/A	N/A	+	+	+	+
CNTS298	2020	Stool	STM	34	F	No	decreased	decreased	normal	normal	-	-	-	+
CNTS305	2020	Stool	STM	37	M	N/A	N/A	N/A	N/A	N/A	-	+	+	+
CNTS306	2020	Stool	STM	52	M	No	decreased	decreased	normal	normal	-	+	+	+
CNTS307	2020	Stool	STM	28	F	No	decreased	normal	increased	decreased	-	+	+	+
CNTS308	2020	Stool	STM	85	F	No	decreased	decreased	normal	normal	-	-	-	+
CNTS309	2020	Stool	SE	1	F	N/A	N/A	N/A	N/A	N/A	+	+	+	+
CNTS310	2020	Stool	STM	48	M	Yes	decreased	increased	increased	decreased	-	-	-	+
CNTS311	2020	Stool	STM	13	M	Yes	decreased	decreased	decreased	normal	-	+	+	+
CNTS318	2020	Stool	STM	0.58	F	Yes	decreased	increased	decreased	increased	-	+	+	+
CNTS325	2020	Stool	STM	68	F	No	decreased	normal	normal	normal	-	+	+	+
CNTS333	2020	Stool	STM	1	M	Yes	decreased	decreased	normal	increased	-	+	+	+
CNTS338	2020	Stool	STM	84	M	No	decreased	decreased	normal	normal	-	-	-	+
CNTS341	2020	Stool	STM	50	M	No	decreased	normal	increased	decreased	-	+	+	+
CNTS360	2021	Stool	STM	1	M	Yes	decreased	decreased	increased	decreased	-	+	+	+
CNTS361	2021	Stool	STM	22	F	No	decreased	decreased	increased	decreased	+	+	+	+
CNTS363	2021	Stool	STM	52	M	Yes	decreased	increased	increased	normal	-	+	+	+
CNTS364	2021	Stool	STM	23	F	No	decreased	increased	normal	normal	-	-	-	+
CNTS367	2021	Stool	SE	46	F	No	decreased	normal	normal	normal	+	+	+	+
CNTS368	2021	Stool	STM	1	M	Yes	decreased	decreased	increased	decreased	-	+	+	+

STM, *Salmonella enterica* Typhimurium; SE, *S. enterica* Enteritidis; M, male; F, female; N/A, Data not available; Hct, hematocrit (normal = 13-18%); Leuk, leukocyte (normal = 5000 – 10000 cells/mm^3); Neutro, neutrophil (normal = 40-74 cells/mm^3) and Lympho, lymphocyte (normal = 19-48 cells/mm^3); +, presence; -, absence

Supplementary Table 2: Demographic and clinical data of hospitalized patients with stool or blood culture positive for *Salmonella enterica* Serovar Typhimurium and Enteritidis (8/8).

Sample ID	Year	Source	Serovar	Age (Year)	Gender	Sepsis	Hct (%)	Leuk. (cells/mm^3)	Neutro. (cells/mm^3)	Lympho. (cells/mm^3)	*spvB*	*sseI*	*sodCI*	*rpoS*
CNTS372	2021	Stool	STM	54	F	Yes	decreased	increased	increased	decreased	-	+	+	+
CNTS377	2021	Blood	SE	71	M	Yes	decreased	normal	increased	decreased	+	+	+	+
CNTS378	2021	Stool	STM	2	M	N/A	N/A	N/A	N/A	N/A	+	+	+	+
CNTS388	2021	Stool	STM	1	F	N/A	N/A	N/A	N/A	N/A	-	+	+	+
CNTS389	2021	Stool	STM	33	F	No	normal	normal	normal	normal	-	+	+	+
CNTS393	2021	Stool	STM	68	M	No	decreased	normal	normal	normal	-	+	+	+
CNTS394	2021	Stool	STM	1	M	N/A	N/A	N/A	N/A	N/A	-	-	-	+
CNTS395	2021	Blood	SE	56	F	Yes	decreased	increased	normal	normal	+	+	+	+

STM, *Salmonella enterica* Typhimurium; SE, *S. enterica* Enteritidis; M, male; F, female; N/A, Data not available; Hct, hematocrit (normal = 13-18%); Leuk, leukocyte (normal = 5000 – 10000 cells/mm^3); Neutro, neutrophil (normal = 40-74 cells/mm^3) and Lympho, lymphocyte (normal = 19-48 cells/mm^3); +, presence; -, absence

Supplementary Table 3: The source, antibiotic resistance pattern, multiple antibiotic resistance (MAR) index, and virulence gene profiles of 6 ESBL-positive *S.* Typhimurium isolates.

Isolate #	Sample ID	Source	Antibiotic resistance pattern	MAR index	Virulence genes profiles
1	CNTS005	blood	S-AMP-TE-DO-CTX-CXM-CIP-FEP-CRO-ATM-CAZ-KZ-NA	0.54	*sseI-sodCI-rpoS*
2	CNTS017	stool	S-AMP-TE-DO-CTX-CXM-FEP-CRO-ATM-CAZ-KZ-DOR-STX	0.54	*sseI-sodCI-rpoS*
3	CNTS028	blood	S-AMP-TE-DO-CTX-CXM-FEP- CRO-ATM-CAZ- KZ-	0.46	*sseI-sodCI-rpoS*
4	CNTS089	stool	S-AMP-TE-DO-CTX-CXM-FEP-CRO-ATM-CAZ-KZ	0.46	*sseI-sodCI-rpoS*
5	CNTS091	stool	S-TE-DO-CTX-CXM-FEP-CRO- ATM-CAZ- KZ-	0.42	*sodCI-rpoS*
6	CNTS098	stool	S-AMP-TE-DO-CTX-CXM-CIP- FEP-CRO-ATM- CAZ- KZ-STX	0.54	*sodCI-rpoS*

Supplementary Table 4: The multiple antibiotic resistance (MAR) index of each *S.* Typhimurium isolate (N = 120)

Sample ID	MAR Index	Sample ID	MAR Index	Sample ID	MAR Index
CNTS005	0.54	CNTS090	0.17	CNTS227	0.17
CNTS012	0.17	CNTS091	0.42	CNTS231	0.21
CNTS016	0.25	CNTS093	0.00	CNTS233	0.00
CNTS017	0.54	CNTS094	0.00	CNTS237	0.00
CNTS019	0.58	CNTS097	0.25	CNTS238	0.13
CNTS023	0.21	CNTS098	0.54	CNTS240	0.21
CNTS028	0.46	CNTS099	0.42	CNTS246	0.54
CNTS029	0.21	CNTS100	0.29	CNTS252	0.21
CNTS031	0.29	CNTS101	0.58	CNTS255	0.29
CNTS032	0.25	CNTS103	0.58	CNTS259	0.21
CNTS037	0.04	CNTS107	0.58	CNTS277	0.13
CNTS039	0.17	CNTS113	0.21	CNTS281	0.17
CNTS040	0.21	CNTS114	0.25	CNTS282	0.21
CNTS041	0.21	CNTS118	0.08	CNTS285	0.13
CNTS045	0.08	CNTS119	0.29	CNTS289	0.29
CNTS046	0.17	CNTS123	0.13	CNTS290	0.04
CNTS047	0.25	CNTS125	0.58	CNTS291	0.25
CNTS048	0.00	CNTS126	0.21	CNTS298	0.21
CNTS049	0.04	CNTS133	0.21	CNTS305	0.17
CNTS050	0.13	CNTS135	0.25	CNTS306	0.17
CNTS053	0.17	CNTS139	0.21	CNTS307	0.04
CNTS055	0.25	CNTS141	0.13	CNTS308	0.04
CNTS056	0.17	CNTS144	0.25	CNTS310	0.04
CNTS058	0.63	CNTS147	0.13	CNTS311	0.21
CNTS060	0.29	CNTS156	0.00	CNTS318	0.25
CNTS064	0.00	CNTS163	0.17	CNTS325	0.25
CNTS065	0.21	CNTS167	0.17	CNTS333	0.04
CNTS068	0.21	CNTS171	0.17	CNTS338	0.13
CNTS069	0.21	CNTS172	0.63	CNTS341	0.08
CNTS070	0.21	CNTS174	0.33	CNTS360	0.13
CNTS071	0.21	CNTS183	0.21	CNTS361	0.21
CNTS073	0.21	CNTS185	0.25	CNTS363	0.08
CNTS075	0.25	CNTS195	0.25	CNTS364	0.04
CNTS078	0.63	CNTS199	0.17	CNTS368	0.08
CNTS080	0.29	CNTS206	0.21	CNTS372	0.08
CNTS081	0.58	CNTS209	0.17	CNTS378	0.08
CNTS083	0.29	CNTS214	0.29	CNTS388	0.08
CNTS085	0.21	CNTS219	0.13	CNTS389	0.08
CNTS086	0.17	CNTS222	0.25	CNTS393	0.17
CNTS089	0.46	CNTS226	0.21	CNTS394	0.21
				**Average**	**0.22**

Supplementary Table 5: The multiple antibiotic resistance (MAR) index of each *S.* Enteritidis isolate (N = 28)

Sample ID	MAR Index
CNTS009	0.08
CNTS010	0.00
CNTS020	0.08
CNTS024	0.04
CNTS030	0.04
CNTS059	0.04
CNTS074	0.08
CNTS105	0.08
CNTS109	0.17
CNTS112	0.25
CNTS115	0.04
CNTS161	0.08
CNTS177	0.04
CNTS182	0.04
CNTS191	0.04
CNTS194	0.13
CNTS200	0.00
CNTS210	0.00
CNTS230	0.00
CNTS251	0.00
CNTS261	0.00
CNTS270	0.00
CNTS272	0.00
CNTS297	0.00
CNTS309	0.00
CNTS367	0.00
CNTS377	0.00
CNTS395	0.00
**Average**	**0.04**
